# Standard- versus intermediate-dose enoxaparin for anti-factor Xa guided thromboprophylaxis in critically ill patients with COVID-19

**DOI:** 10.1186/s12959-021-00337-z

**Published:** 2021-11-15

**Authors:** David Oliver Hamilton, Alexander Main-Ian, Jessie Tebbutt, Maya Thrasher, Alicia Waite, Ingeborg Welters

**Affiliations:** 1 Critical Care Department, Royal Liverpool University Hospital, Prescot St, Liverpool, UK; 2 Institute of Life Course and Medical Sciences, University of Liverpool, Liverpool, UK

## Abstract

The prevalence of venous thromboembolism (VTE) is high in critically ill patients with COVID-19. Dosing of Low Molecular Weight Heparin (LMWH) for thromboprophylaxis in patients with severe COVID-19 is subject to ongoing debate.

In this brief report, we describe our study where we retrospectively examined the efficacy of standard- versus intermediate-dosing of enoxaparin in attaining and maintaining accepted prophylactic levels of anti-Factor Xa (anti-FXa) in critically ill patients with COVID-19.

We collected data for all patients with confirmed COVID-19 who were treated with enoxaparin for thromboprophylaxis in a single Intensive Care Unit (ICU) in the United Kingdom between 31st March and 16th November 2020. Standard-dose of enoxaparin was 40 mg subcutaneously once daily for patients with normal renal function and body weight between 50 and 100 kg; the intermediate-dose was 40 mg subcutaneously twice daily. Anti-FXa peak concentrations between 0.2-0.4 IU/ml were considered appropriate for thromboprophylaxis.

Age, sex, weight, Body Mass Index, APACHE II score, ICU length of stay, initial P/F ratio and creatinine were not statistically significantly different between standard- and intermediate-dose thromboprophylaxis cohorts. In the standard-dose group, the median initial anti-FXa level was 0.13 (interquartile range 0.06-0.18) compared to 0.26 (0.21-0.33) in the intermediate-dose cohort (*p* < 0.001). On repeated measurement, in the standard dose cohort, 44 of 95 (46%) anti-FXa levels were < 0.2 IU/ml compared with 24 of 132 (18%) levels in the intermediate-dose cohort even after dose-adjustment. There was one radiologically confirmed pulmonary embolism (PE) on computed tomography pulmonary angiogram during hospital admission in each cohort.

Our study supports starting intermediate-dose thromboprophylaxis for critically ill patients with COVID-19 to achieve anti-FXa levels in the accepted thromboprophylactic range although further study is required to investigate whether anti-FXa guided thromboprophylaxis is safe and effective in reducing the incidence of VTEs in critically ill patients with COVID-19.

## Brief report

The prevalence of venous thromboembolism (VTE) is high in critically ill patients with COVID-19 [1]. Dosing of Low Molecular Weight Heparin (LMWH) for thromboprophylaxis in patients with severe COVID-19 is subject to ongoing debate. Several guidelines suggest the use of an ‘intermediate’ dose of LMWH instead of standard dosing [1–3]. Anti-Factor Xa (anti-FXa) monitoring has been used to titrate LMWH doses for prophylactic or therapeutic anticoagulation in critically ill patients [4]. Results of a recent randomised clinical trial do not support routine use of intermediate-dose prophylactic anticoagulation in patients with COVID-19, however anti-FXa levels were not measured in this study [5].

In this study, we retrospectively examined the efficacy of standard- versus intermediate-dosing of enoxaparin in attaining and maintaining accepted prophylactic levels of anti-FXa in critically ill patients with COVID-19.

We collected data for all patients with confirmed COVID-19 who were treated with enoxaparin for thromboprophylaxis in the Royal Liverpool University Hospital Intensive Care Unit (ICU) between 31st March and 16th November 2020 who had at least one anti-FXa level measured. Patients on therapeutic anticoagulation, patients transferred in from other ICUs or those deemed to have an incidental diagnosis of COVID-19 were excluded.

Standard-dose of enoxaparin was 40 mg subcutaneously once daily for patients with normal renal function and body weight between 50 and 100 kg; the intermediate-dose was 40 mg subcutaneously twice daily. From 21st April 2020, our institution changed to intermediate-dose LMWH, with further dose adjustments guided by anti-FXa levels, for prophylaxis as the standard of care after early evidence for COVID-19-associated coagulopathy emerged [1, 6–8]. This change allowed the comparison of two cohorts: patients receiving standard-dosing and patients receiving intermediate-dosing. There were no further changes to the thromboprophylaxis protocol through the study period.

Anti-FXa levels were measured after at least three consecutive doses of enoxaparin (Inhixa, Techdow Pharmaceuticals, Shenzhen, China). Peak anti-FXa levels were obtained four hours after dose administration and quantified using citrated plasma (Sodium Citrate S-Monovette, Sarstedt, North Carolina, USA) in a chromogenic assay (HemosIL Liquid Anti-Xa, Instrumentation Laboratory, Bedford, USA). The test is accurate to a detection limit < 0.04 IU/ml and has a coefficient of variation value of < 6% [9]. It should be noted that dextran is included in the reaction mixture used in both cohorts and no blood tubes used contained the Citrate Theophylline Adenosine Dipyridamole (CTAD) mixture.

Anti-FXa concentrations between 0.2-0.4 IU/ml were considered appropriate for thromboprophylaxis [1, 4, 10, 11]. Dose adjustments were made at clinicians’ discretion if levels were not in range. Anti-FXa monitoring was repeated after dose adjustments or weekly if levels were within range.

Data were collected retrospectively from routine clinical data until death, ICU discharge or day 14 of ICU admission. Approval for the study was given by the hospital’s Clinical Effectiveness department. Data were analysed using R 3.6.3 for MS Windows. Comparison between standard- and intermediate-dose thromboprophylaxis was made using Fisher’s exact test for categorical data and Mann-Whitney U test for numeric data. *P* values < 0.05 were considered significant.

Age, sex, weight, Body Mass Index, APACHE II score, ICU length of stay, initial P/F ratio and creatinine were not statistically significantly different between standard- and intermediate-dose thromboprophylaxis cohorts (Table 1). Inpatient mortality was 43.1%, 11 out of 23 (47.8%) patients in the standard-dose cohort and 14 out of 35 (40.0%) in the intermediate-dose cohort.

**Table 1:** Characteristics and results for standard-dose cohort (left) and intermediate-dose cohort (right)

	Standard-dose			Intermediate-dose			***p***-value
	n	Median	IQR	n	Median	IQR	
Age (years)	23	58	[46.5 - 63.5]	35	53	[45.5 - 67]	*p* = 0.874
Weight (kg)	23	90	[79 - 96.5]	35	86	[76.5 - 98]	*p* = 0.427
Body Mass Index	23	30.3	[25.8 - 32.0]	35	29	[24.8 - 31.3]	*p* = 0.276
APACHE II Score	20	12.5	[11.8 - 15.3]	32	12.5	[10 - 16.3]	*p* = 0.699
Critical Care admission (days)	23	19	[12 - 22]	34	15	[9.3 - 21]	*p* = 0.353
P/F Ratio (kPa)	23	19.4	[15.3 - 26.9]	35	18.5	[15.4 - 25.3]	*p* = 0.674
PEEP (cmH2O)	22	11	[8.5 - 12.8]	34	10	[7.5 - 10]	***p*** **= 0.033**
Noradrenaline (mcg/kg/min)	23	0	[0 - 0.01]	35	0	[0 - 0]	*p* = 0.053
Creatinine (micromol/L)	23	74	[57 - 99]	35	64	[53 - 81]	*p* = 0.097
Estimated fluid balance (ml)	23	2800	[1650 - 6250]	33	700	[0 - 1900]	***p <*** **0.001**
First Anti-Factor Xa level (IU/ml)	23	0.13	[0.06 - 0.18]	35	0.26	[0.21 - 0.33]	***p <*** **0.001**
Starting daily dose enoxaparin (mg)	23	40	[40 - 40]	35	80	[80 - 80]	***p <*** **0.001**
Dose by weight (mg/kg)	23	0.44	[0.42 - 0.51]	35	0.93	[0.82 - 1.04]	***p <*** **0.001**
Adjustment period (days)	16	2	[0 - 3]	34	0	[0 - 0.8]	***p*** **= 0.003**

*IQR* Inter-quartile range, *PEEP* positive end expiratory pressure.

Bold text highlights statistical significance.

In the standard-dose group, the median initial anti-FXa level was 0.13 (interquartile range 0.06-0.18) compared to 0.26 (0.21-0.33) in the intermediate-dose cohort (*p* < 0.001).

Ninety-five anti-FXa levels were measured in the standard-dose cohort. Forty-four (46%) of these measurements were below 0.2 IU/ml compared with 24 of 132 (18%) levels in the intermediate-dose cohort. Ten of the 58 (17%) patients included had only one anti-Xa level measured, hence the majority of patients had repeated measurements to allow assessment on maintaining appropriate anti-FXa levels. Patients with only one measurement had either died, been changed to therapeutic anticoagulation or were discharged early after admission.

**Fig. 1:**
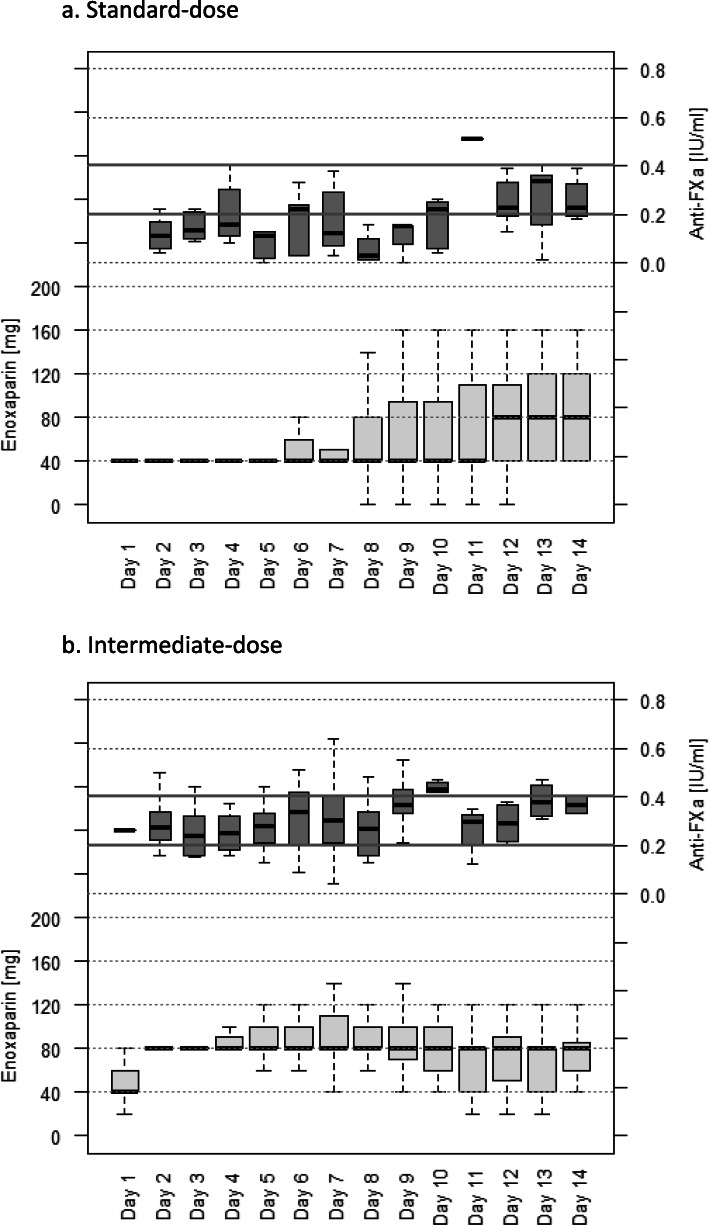
Anti-Factor Xa levels and total daily enoxaparin dose administered across for the first 14 days of Intensive Care Unit (ICU) admission for standard-dose (**a**) and intermediate-dose cohorts (**b**). Doses were adjusted throughout admission at clinicians’ discretion. Day 1 reflects admission day to ICU, therefore depending on the time of admission, patients in the intermediate-dose cohort received either two or only one dose of enoxaparin

Figure 1a&b show the anti-FXa levels for standard and intermediate dosing for the first 14 days of ICU admission. In Fig. 1a&b, Day 1 represents admission to ICU, and shows a high variation in the dose of enoxaparin administered as it was typically prescribed at 06:00 and 18:00 and therefore the time of admission in the day would affect the dose received by the patient.

In each cohort, one patient had pulmonary embolism (PE) confirmed on computed tomography pulmonary angiogram during hospital admission. In total, 18 patients required escalation to therapeutic anticoagulation (standard-dose cohort: 6 patients, intermediate-dose cohort: 12 patients), for the following reasons: empiric PE treatment after deterioration (10 patients, four of whom later had imaging, all of which was negative); atrial fibrillation (three patients); confirmed PE (two patients); extracorporeal membrane oxygenation (two patients) and renal replacement therapy (one patient). There were no major bleeding events as defined by the International Society on Thrombosis and Haemostasis criteria [12].

These data suggest that intermediate-dose is more likely to result in accepted anti-FXa levels sufficient for thromboprophylaxis compared to standard-dosing. This implies that critically ill patients with COVID-19 may be at risk from VTE or immunothromboses if treated with standard-dose enoxaparin. This may be due to the high level of circulating acute phase reactants, such as fibrinogen, neutrophil extracellular traps and histones [1]. It may also be a feature of critical illness itself. Anti-FXa levels are markedly lower in critically ill patients with COVID-19 on standard-dosing compared to ward-based patients [11]. Pre-pandemic literature also suggests that conventional standard-dose LMWH was associated with low anti-FXa levels in critically ill patients [13, 14].

There are several limitations to this study: firstly, the results of this small single-centre study may not be applicable in different settings or to the use of other LMWH or anti-FXa testing procedures. Larger prospective studies are required to confirm that intermediate-dosing of LMWH is more reliable in attaining satisfactory anti-FXa levels for thromboprophylaxis. Secondly, adjusting thromboprophylaxis to target anti-FXa levels has not been shown to improve clinical outcomes in critical illness [15]; and there is no universally accepted target range. Further research is required to assess the efficacy, safety and reproducibility of anti-FXa guided thromboprophylaxis.

In conclusion, our study supports the use of intermediate-dose thromboprophylaxis for critically ill patients with COVID-19 to achieve anti-FXa levels considered appropriate for thromboprophylaxis. However, monitoring of anti-FXa levels has not been shown to improve clinical outcomes in critical illness. The efficacy of intermediate-dose thromboprophylaxis for prevention of venous thromboembolism (VTE) remains uncertain, and larger clinical trials are needed to investigate whether anti-FXa guided thromboprophylaxis is safe and effective in reducing the incidence of VTEs in critically ill patients with COVID-19.

## Data Availability

Availability of data depends on approval by Liverpool University Hospitals NHS Foundation Trust.
